# Urinary Biomarkers of Whole Grain Wheat Intake Identified by Non-targeted and Targeted Metabolomics Approaches

**DOI:** 10.1038/srep36278

**Published:** 2016-11-02

**Authors:** Yingdong Zhu, Pei Wang, Wei Sha, Shengmin Sang

**Affiliations:** 1 Laboratory for Functional Foods and Human Health, Center for Excellence in Post-Harvest Technologies, North Carolina Agricultural and Technical State University, North Carolina Research Campus, Kannapolis, NC, USA; 2 Bioinformatics Services Division, University of North Carolina at Charlotte, North Carolina Research Campus, Kannapolis, NC, USA

## Abstract

Mounting evidence suggests that whole grain (WG) intake plays an important role in chronic disease prevention. However, numerous human studies have failed to produce clear-cut conclusions on this topic. Here, a combination of non-targeted and targeted metabolomics approaches, together with kinetic studies, was used to investigate biomarkers of WG wheat intake and further explore the diet-disease associations. Via these integrated approaches, forty-one compounds were identified as the most discriminating endogenous metabolites after WG versus refined grain (RG) wheat bread consumption. The corresponding biological assessment of these endogenous changes suggests that, in contrast to RG consumption, WG wheat consumption may facilitate antioxidant defense systems and moderate the risk factors of cancer, cardiovascular diseases, and other chronic diseases. A panel of urinary markers consisting of seven alkylresorcinol metabolites and five benzoxazinoid derivatives as specific biomarkers, as well as five phenolic acid derivatives, was also established to cover multiple time points and longer time periods for correctly and objectively monitoring WG wheat intake. Through these findings, we have established a comprehensive biomarker pool to better assess WG wheat consumption, and to monitor the endogenous changes that are linked to health effects of WG wheat consumption.

Metabolomics is the comprehensive analysis of all metabolites in a biological system^1^, and has been applied in various areas to quantitatively assess biochemical fluxes and metabolites that are indicative of unusual biological or environmental perturbations^2^. Metabolic analysis is typically categorized into two complementary methods: targeted and non-targeted. The non-targeted analysis measures all small molecules including endogenous and exogenous metabolites in biological samples and then identifies potential and putative metabolites of interest for further tests. In contrast, a targeted approach mainly focuses on the identification and quantification of selected metabolites^3^. Recently, nutrimetabolomics, which refers to metabolomics in nutritional sciences, has been developed to explore the complex relationships between the dietary consumption and health outcomes in animals and to also investigate the endogenous changes after dietary intake^4^.

Whole grains (WG) contain endosperm, germ, and bran, in contrast to refined grains (RG) which have the germ and bran removed during the milling process^5^. Modern nutritional epidemiology indicates that WG intake, but not RG intake, is inversely associated with the risk of major chronic diseases, such as cancer^6^,^7^,^8^, cardiovascular diseases (CVD)^7^,^9^,^10^, type 2 diabetes^6^,^7^,^11^, and obesity^12^,^13^,^14^. However, the outcomes of large-scale prospective cohort studies or human intervention studies testing the causality of these relationships have often proved inconclusive or have failed to demonstrate causality of cancers^15^,^16^, CVD^17^,^18^, and diabetes^18^,^19^. Some metabolomics studies have tried to predict the diet-disease associations by interpreting the putative links between the risk factors of diseases and certain endogenous changes^20^,^21^. However, in these studies only spot urine or plasma samples were collected, and the determined endogenous metabolites alterations were limited as well, which may have clouded the diet-disease association. The accumulation and excretion of the postprandial metabolites can be monitored by kinetic studies^22^,^23^. Therefore, non-targeted metabolomics approaches coupled with kinetic analysis in a diet-controlled trial may enhance the determination of changes in numerous endogenous metabolites and thus facilitate the estimation for health effects of WG intake.

It is challenging to accurately measure WG intake with the traditional self-assessment approaches typically used in large observational studies such as food journals and food frequency questionnaires due to inherent limitations^24^. Moreover, translation of food intake into energy, nutrients, and bioactive food components is heavily dependent on food composition tables^25^. Measurement errors associated with assessment methods further compound the problem of dietary estimates and may also obscure the diet-disease associations. For this reason, there is a pressing need for dietary biomarkers to better capture exposure. To date, WG alkylresorcinols (ARs) and their metabolites 3,5-dihydroxybenzoic acid (3,5-DHBA) and 3-(3,5-dihydroxyphenyl)-1-propanoic acid (3,5-DHPPA) have been developed as major potential exposure biomarkers for WG wheat and rye intake in epidemiological studies^26^,^27^. In addition, benzoxazinoid (BX) derivatives, such as 2-hydroxy-N-(2-hydroxyphenyl)acetamide (HHPAA) and N-(2-hydroxyphenyl)acetamide (HPAA), have recently been identified as alternative biomarkers to discriminate WG wheat and rye consumers from control group^28^. However, there are limitations for ARs, BXs, or their metabolites when as single use exposure biomarkers of WG wheat and rye intake in cohort studies. ARs are merely short- to medium-term biomarkers of intake of WG wheat and rye, with estimated apparent half-lives and absorption half-lives in plasma at ~5 h and 6–8 h, respectively^29^. The poor/moderate reproducibility for AR metabolites, 3,5-DHBA and 3,5-DHPPA^30^, may also limit the use of single measurements of these metabolites in cohort studies, and BXs are also found in WGs of maize, wild barleys, and other human plant food^31^. Therefore, the discovery of more specific biomarkers for WG consumption would give birth to a better assessment of compliance in large-scale human studies.

Whole wheat is one of the top ten largest-selling baked goods in supermarkets in USA^32^. Determination of specific WG wheat biomarkers helps to better assess whole wheat consumption in epidemiological studies. In the present study, a non-targeted metabolomics approach was applied to analyze all metabolites, including dietary exposures and endogenous biomarkers, in urine samples collected from WG wheat bread- and RG wheat bread-consumers, and a targeted metabolomics approach was utilized to further investigate the metabolism of specific WG wheat phytochemicals. Concomitantly, kinetic studies were applied with both approaches. The objective of this study was to understand the impact of dietary WG wheat intake on the endogenous metabolome. Another aim was to generate new insights into biological mechanisms underlying the health-related effects of WG wheat consumption on major chronic diseases, including cancers, CVD, and diabetes. Efforts were also made to identify more specific biomarkers that precisely define dietary exposures and thus provide better estimates of diet-disease associations.

## Results

### Non-targeted metabolomics: endogenous and exogenous urinary metabolites

#### Data analysis

The metabolomics data was analyzed by three different Orthogonal Partial Least Squares (OPLS) analyses, including 1) regular OPLS discriminant analysis (OPLS-DA), 2) OPLS effect projections (OPLS-EP), an OPLS analysis for paired data, and 3) OPLS-DA based batch processing analysis, an OPLS method for multiway data.

**Figure 1:**
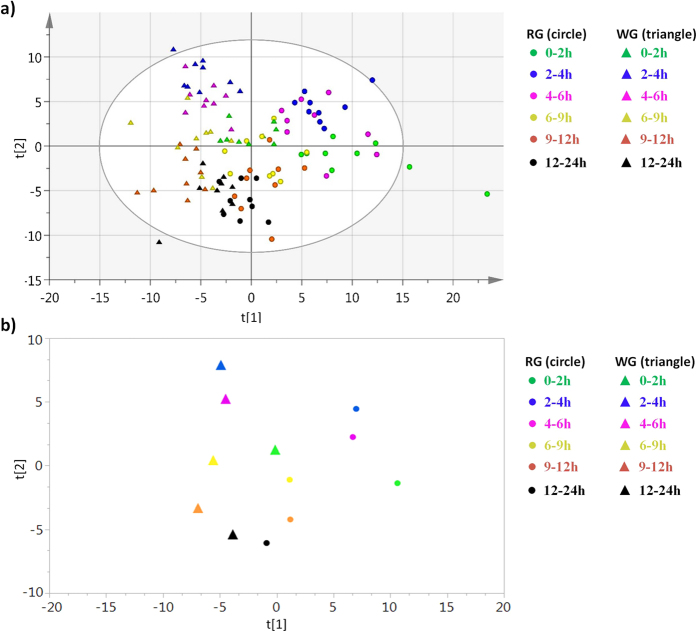
OPLS-DA separates RG and WG samples. (**a**) OPLS-DA score scatter plot for the separation of RG samples (circles) and WG samples (triangles) at each interval. The ellipse marked the 95% Hotelling T^2^ control chart, showing possible outliers. (**b**) The centroid of RG samples (circles) and WG samples (triangles) at each interval presented in the same color scheme as (**a**).

**Figure 2:**
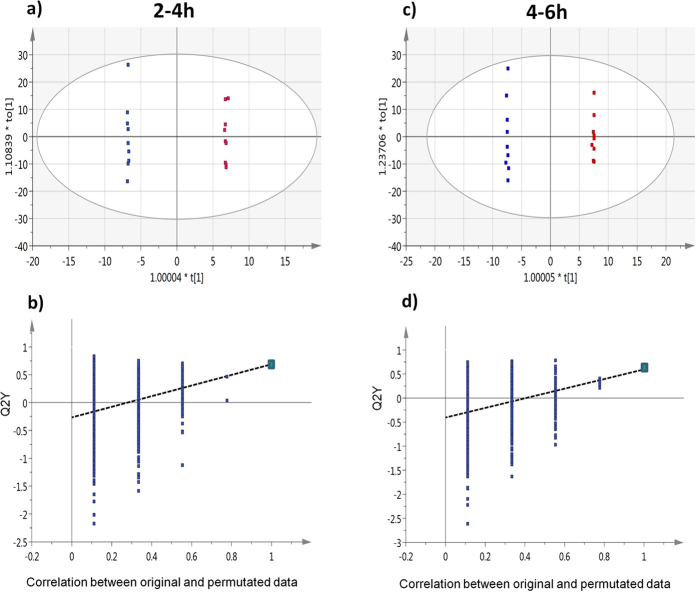
OPLS-DA plots for the separation between RG samples and WG samples at selected time points. OPLS-DA score scatter plots of RG samples (blue squares) and WG samples (red squares) at intervals of 2–4 h (**a**) and 4–6 h (**c**). The ellipse marked the 95% Hotelling T^2^ control chart, showing possible outliers. (**b**,**d**) are the OPLS-DA validation plots for the separation observed in (**a**,**c**), respectively. Q^2^Y from the original data and permutated data are represented as green and blue squares, respectively. As shown, Q^2^Y from the original data was found to be higher than at least 95% of the Q^2^Y generated from the 999 permutated data sets.

The regular OPLS-DA was applied to investigate differences in the urinary metabolomics profiles between the WG and RG samples. All of the intervals were included in one analysis to examine the overall pattern of the data sets (Fig. 1a). Altogether, 579 metabolites were profiled. Between the two interventions, the best separations were observed from urine samples collected at 0–2 h, 2–4 h, and 4–6 h followed by samples at 6–9 h and 9–12 h (Fig. 1a,b). The data from urine samples collected at 12–24 h were overlapped between the two treatments (Fig. 1a,b). To verify the validity of the separation at each interval between the WG and RG groups, OPLS-DA was further performed at each interval separately, and then permutation-based validation was used to determine if the separation was statistically valid. As a result, only separations at 2–4 h (Fig. 2a) and 4–6 h (Fig. 2c) passed permutation-based validation and were found to be valid. The values of R^2^Y = 1 and Q^2^Y = 0.688 for interval of 2–4 h, and R^2^Y = 1 and Q^2^Y = 0.596 for interval of 4–6 h, indicate good model fit and predictive ability. The predictive ability of this model was found to be better than at least 95% of the models built using the 999 permutated datasets, therefore the probability that the separations at 2–4 h and 4–6 h were due to chance was less than 5%, i.e. the separations were found to be significant (Fig. 2b,d).

**Figure 3:**
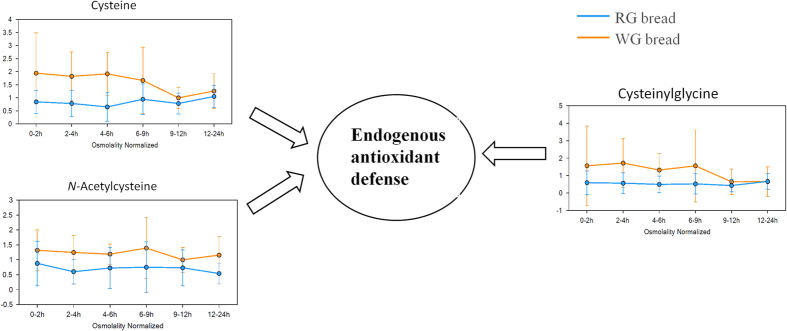
Alterations in thiol-related metabolites in the RG (blue) and WG (red) groups. Box plots showed accumulations in cysteine, NAC, and cysteinylglycine in the WG group compared to the RG group. The y-axis indicates area counts/osmolality (mOsm/kg). Data are expressed as mean ± SEM. NAC, *N*-acetylcysteine.

**Figure 4:**
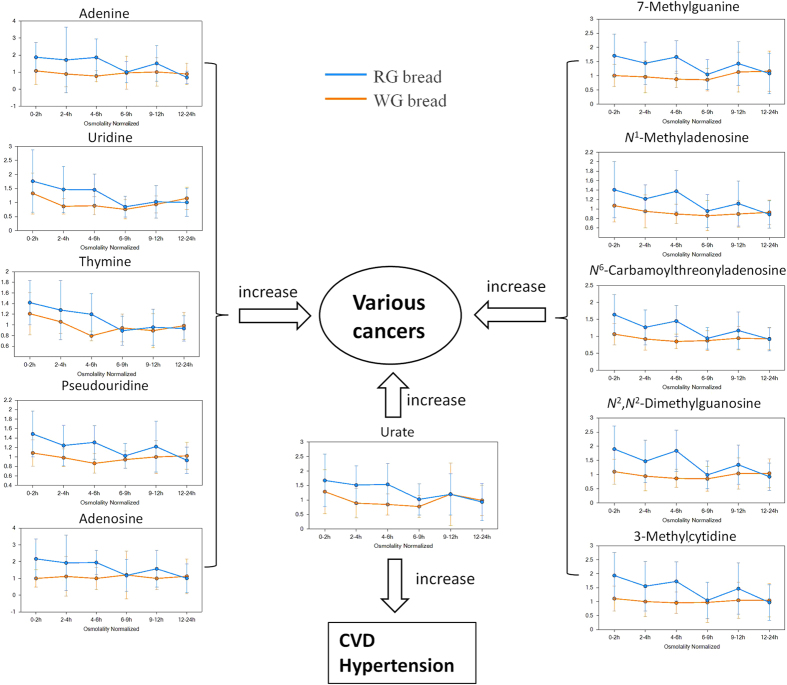
Abundance differences and risk predictions in nucleotide pools in the RG (blue) and WG (red) groups. Box plots showed decreases in adenine, adenosine, 3-methylcytidine, uridine, thymine, Pseudouridine, urate, 7-methylguanine, *N*^1^-methyladenosine, *N*^6^-carbamoylthreonyladenosine, and *N*^2^,*N*^2^-dimethylguanosine in the WG group compared to the RG group. The y-axis indicates area counts/osmolality (mOsm/kg). Data are expressed as mean ± SEM.

**Figure 5:**
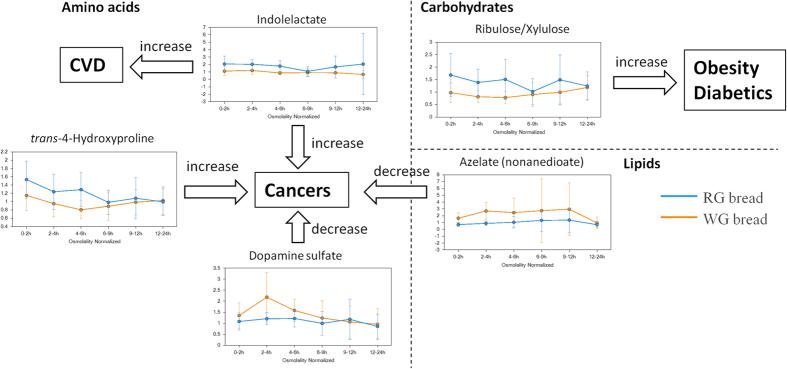
Abundance differences and risk predictions in metabolites of amino acids, lipids, and carbohydrates in the RG (blue) and WG (red) groups. The y-axis indicates area counts/osmolality (mOsm/kg). Data are expressed as mean ± SEM.

Because of the repeated measures design, OPLS-EP, a multivariate data analysis method specifically designed for paired data^33^, was applied to compare WG and RG samples at respective intervals of 2–4 h and 4–6 h. The model fit and predictive ability of the OPLS-EP models for 2–4 h (R^2^Y = 1, Q^2^Y = 0.792) and 4–6 h (R^2^Y = 0.96, Q^2^Y = 0.725) were found to be even better than OPLS-DA. To identify the most discriminating urinary metabolic differences after WG versus RG consumption, a cut-off of Variability Influence on Projection (VIP) >1.5 in OPLS-EP was applied to the two intervals of 2–4 h and 4–6 h, separately. The metabolites with VIP >1.5 and *p* ≤ 0.05 at the 2–4 h and/or 4–6 h intervals were considered as the most discriminating metabolites. On the basis of clustered mass features, exact masses for in-source fragments, and experimental MS/MS fragmentations, 62 compounds were identified (Table 1). Their super/subpathways and statistical parameters including fold changes, VIP scores from OPLS-EP, and *p* and *q* values from repeated measures ANOVA are summarized in Table 1. Meanwhile, kinetic curves for 62 metabolites in the two groups were generated as box plots (Figs 3,4 and 5 and Supplementary Fig. 1). These discriminating metabolites were classified into two groups: endogenous metabolites and food-derived metabolites (exogenous metabolites). Among them, 41 metabolites are endogenous with seven elevated and two decreased at 2–4 h of interval, two elevated and 25 decreased at 4–6 h of interval, and five decreased at both 2–4 h and 4–6 h in WG samples; and 21 metabolites are food-derived with six increased at 2–4 h of interval, three decreased at 4–6 h of interval, and nine elevated and three decreased at both 2–4 h and 4–6 h in WG samples (Table 1).

To explicitly include the time dimension in the analysis, OPLS-DA based batch processing analysis was applied. Batch processing analysis has been used to analyze multiway data, including the analysis of time series metabolomic data^34^,^35^. This analysis operates on two levels. At the lower level, OPLS regression was performed to study trajectories of samples along the time dimension. At the upper level (batch level), OPLS-DA was performed to differentiate WG and RG samples when all of the time points were considered. The trajectories were not found to be markedly different between WG and RG samples (data not shown). This is not unexpected, because the lower level OPLS analysis was intended to find metabolic variations related to time, and was not aimed to differentiate WG and RG samples. On the other hand, the upper level OPLS-DA, which was aimed to provide differentiation, was able to separate WG and RG samples (Supplementary Fig. 2a). However, the separation did not pass permutation-based validation (Supplementary Fig. 2b). Altogether, the batch analysis suggests that there was some overall difference between WG and RG samples when all of the time points were considered. However, the overall difference was not strong enough to reach statistical significance. The significant difference between WG and RG was only observed at intervals of 2–4 h and 4–6 h, separately, as suggested by regular OPLS-DA (Fig. 2) and OPLS-EP (Table 1).

#### Changes of endogenous metabolites

Non-targeted metabolomics is a powerful tool to determine the changes in endogenous metabolites between two interventions. The urinary endogenous changes determined in the present study could be classified into three groups as follows.

##### Accumulation of cysteine and related thiol compounds within the WG group

When the WG group was compared to the RG group, significant increases in urinary cysteine were seen across the intervals (0–2, 2–4, 4–6, 6–9, 9–12, and 12–24 h) of the analysis (Table 1 and Fig. 3), regardless of the presence of cysteine in the body endogenously and exogenously^36^. Although cysetine-related thiol compounds, *N*-acetylcysteine (NAC), and cysteinylglycine, did not pass the cut-off VIP >1.5 in OPLS-EP analysis, their VIP scores were greater than 1.2 at 2–4 h after WG versus RG consumption (Supplementary Table 1). The excretion of urinary NAC in the WG group was found to be much higher than that in the RG group during all intervals (Supplementary Table 1 and Fig. 3). Elevated cysteinylglycine in the WG group was also observed in 12 h after intake compared to the RG group (Supplementary Table 1 and Fig. 3). These observations suggest that WG consumption accumulates cysteine and related thiol compounds in the body.

##### Significant differences with regards to nitrogen balance between WG and RG groups

It is largely accepted that nitrogen balance reflects both protein and energy intake from the diet^37^. The WG consumers exhibited significant decreases in several urinary metabolites related to nucleosides following WG wheat consumption compared to the RG group (Table 1 and Fig. 4). As shown, the levels of adenine, uridine, thymine, and adenosine in the WG group were reduced at the 0–2, 2–4, and 4–6 h intervals (Fig. 4). Moreover, decreases in pseudouridine (Pseu) in the WG group were extended to 9–12 h when compared to the control group (Fig. 4). Likewise, the excretion of 7-methylguanine (m7Gua) in the WG consumers was reduced at 12 h after consumption (Fig. 4). The WG consumption also decreased the levels of *N*^1^-methyladenosine (m1A), *N*^6^-carbamoylthreonyladenosine (t6A), *N*^2^,*N*^2^-dimethylguanosine (m22G), and 3-methylcytidine, from 0–2 to 9–12 h (Table 1 and Fig. 4). Decreases in urate in the WG group were also found from 0–2 to 6–9 h after intake (Fig. 4). In addition to metabolites related to nucleosides, WG intake also inversely correlated with metabolites of some amino acids, such as tryptophan and proline (Table 1 and Fig. 5). Decreases in indolelactate in the WG group were seen across all intervals (Table 1 and Fig. 5). Decreased levels of *trans*-4-hydroxyproline in the WG consumers were observed from the starting interval (0–2 h) to the late interval (9–12 h) (Fig. 5). On the other hand, the WG group displayed significant increases in dopamine (DA) sulfate in the WG consumers in the early intervals (<9 h) after consumption (Fig. 5).

##### Metabolism of lipids and carbohydrates

The WG consumption displayed an increase in urinary azelaic acid (AzA) in 12 h after intake (Table 1 and Fig. 5). When the samples of the WG and RG groups were compared, a decrease in ribulose/xylulose in the WG consumers were also observed (Table 1 and Fig. 5).

#### Changes of exogenous metabolites

With regard to food-derived metabolites, significant increases in 3,5-DHBA, and HPAA sulfate in the WG group (Table 1 and Supplementary Fig. 1) were attributed to wheat phytochemicals ARs and BXs^38^,^39^. Other altered compounds linked to WG consumption are phenolic acids and related metabolites, such as ferulic acid (FA) sulfate, 1,2,3-benzenetriol sulfates degraded from gallic acid^40^, 3-methoxycatechol sulfate derived probably from 3-methylgallic acid^40^, 3-methylcatechol^41^, and 4-acetylphenol^42^ (Table 1 and Supplementary Fig. 1). Tartarate, derived from food preparation^43^, also exhibited significant increases in the WG group in relation to the RG group (Table 1 and Supplementary Fig. 1).

### Targeted metabolomics: urinary metabolites of WG wheat phytochemicals

#### Identification of urinary metabolites of WG wheat phytochemicals

**Figure 6:**
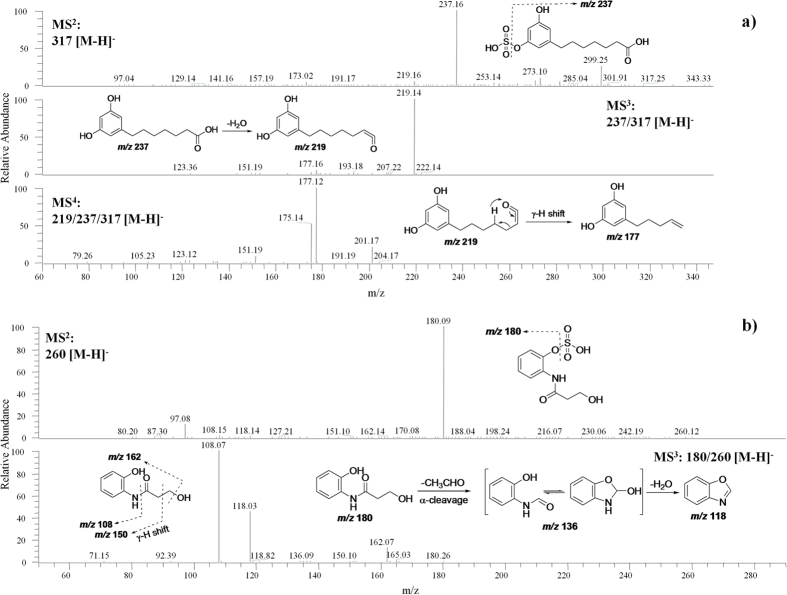
Structural identification of new metabolites. LC-MS^n^ (*n* = 2–4) spectra of DHPHTA sulfate (**a**) and HHPPA sulfate (**b**). DHPHTA, 7-(3,5-dihydroxyphenyl)heptanoic acid; HHPPA, 3-hydroxy-*N*-(2-hydroxyphenyl)propanamide.

The identification of metabolites that are not included in mass spectral libraries is the current bottleneck in non-targeted LC-MS-based metabolomics studies^2^. Most of the metabolites originated from specific components in food are not commercially available and are unlikely to be included in the libraries of non-targeted metabolomics. These specific metabolites can be investigated by targeted approach based on the chemical properties of their known sources^2^. WG wheat is a rich source of various phytochemicals such as ARs, BXs, phytosteroids, lignans, and phenolic acids^44^. In the present study, a targeted LC-tandem MS in data-dependent acquisition was applied to explore the metabolites of these specific wheat phytochemcials. Urinary metabolites of phytochemicals were identified by comparison of their retention times and fragmentation patterns with standards or data in literature, eventually resulting in the discovery of 26 metabolites (Table 2). Among them, 20 metabolites were identified by targeted approaches only. These metabolites were chemically categorized into five groups: AR metabolites, BX derivatives, phenolic acid derivatives, phytosterol-related compounds, and metabolites of lignans. Particularly, three metabolites, 7-(3,5-dihydroxyphenyl)heptanoic acid (DHPHTA) sulfate (**7**), 3-hydroxy-*N*-(2-hydroxyphenyl)propanamide (HHPPA) sulfate (**13**), and glycochenodeoxychol-5-en-24-oic acid glucuronide (**25**) were identified as new metabolites (Table 2, and Fig. 6).

##### AR metabolites

**Figure 7:**
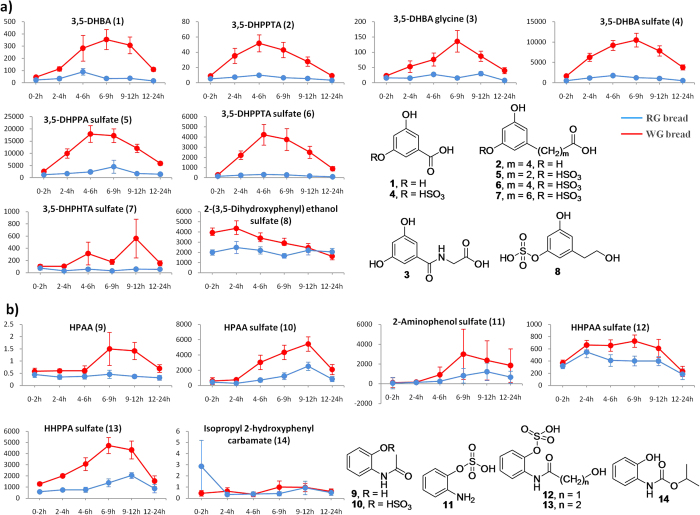
Kinetics study. Kinetics curves and structures of the main metabolites derived from wheat phytochemicals, alkylresorcinols (**a**) and benzoxazinoids (**b**), after RG- and WG bread consumptions at different intervals (0–2 h, 2–4 h, 4–6 h, 6–9 h, 9–12 h, and 12–24 h). The y-axis indicates peak area/osmolality (mOsm/kg). Data are expressed as mean ± SEM.

The urinary excretion of AR metabolites, 3,5-DHBA (**1**), 3,5-DHPPTA (**2**), and 3,5-DHBA glycine (**3**) (Table 2 and Fig. 7), was confirmed by comparing their retention times and MS/MS fragmentations with standards. The presence of AR metabolite 3,5-DHBA has also been granted by the non-targeted metabolomics approach above. Metabolites **4–6** were identified as 3,5-DHBA sulfate (**4**), 3,5-DHPPA sulfate (**5**), and 3,5-DHPPTA sulfate (**6**) (Table 2 and Fig. 7), as reported in a previous study^22^. Metabolite **7** had a molecular ion at *m/z* 317 (237+80) [M−H]^−^ under negative ESI/MS mode, which was 28 units higher than that of 3,5-DHPPTA sulfate (**6**), indicating that two more –CH_2_ groups are present in the structure of metabolite **7** than in **6**. Similar to **6**, metabolite **7** had fragment ions at *m/z* 219 [M−sulfate−H_2_O−H]^−^, *m/z* 193 [M−sulfate−CO_2_−H]^−^, and *m/z* 177 [M−sulfate−HOAc−H]^−^ (corresponding to a neutral loss of HOAc from aglycone through γ-H shift) in its MS^3^ spectrum (Fig. 6a). Further breakdown of the daughter ion at *m/z* 219 produced the major fragment at *m/z* 177 in MS^3^ spectrum of **7**, which was formed by McLafferty rearrangement via a neutral loss of CH≡COH (Fig. 6a). All of these spectra features suggest that metabolite **7** is 7-(3,5-dihydroxyphenyl)heptanoic acid (DHPHTA) sulfate (Fig. 6a), a new metabolite of ARs. In addition, metabolite **8** had a major MS^3^ fragment ion at *m/z* 123 [M−sulfate−CH_2_OH−H]^−^, suggesting that **8** is 3,5-dihydroxyphenylethanol sulfate (Table 2), a known metabolite of WG rye in human^45^.

##### BX derivatives

The structure of *N*-(2-hydroxyphenyl)acetamide (HPAA) (**9**) (Table 2 and Fig. 7) was confirmed by comparison of its retention time and MS/MS fragmentation pattern with those of the authentic standard. HPAA sulfate (**10**), 2-aminophenol sulfate (**11**), and HHPAA sulfate (**12**) (Table 2 and Fig. 7) have been reported as biomarkers of WG rye bread exposure^45^. Metabolite **13** had a molecular ion at *m/z* 260 (180+80) [M−H]^−^, which was 14 units higher than that of **12** (Table 2), indicating that one more –CH_2_ group appears in the structure of metabolite **13** than in metabolite **12**. Similar to **12**, **13** had daughter ions at *m/z* 162 [M−sulfate−H_2_O−H]^−^, *m/z* 150 [M−sulfate−HCHO−H]^−^ (neutral loss of HCHO via γ-H shift), *m/z* 118 [M−sulfate−CH_3_CHO−H_2_O−H]^−^ (formed by loss of CH_3_CHO via α-cleavage of amide followed by loss of H_2_O after cyclization of intermediate), and *m/z* 108 (corresponding to the breakdown of amide bond) in its MS^3^ spectrum (Fig. 6b). These spectra features demonstrated that metabolite **13** is 3-hydroxy-*N*-(2-hydroxyphenyl)propanamide (HHPPA) sulfate (Fig. 6b), a new derivative of BXs. Isopropyl-2-hydroxyphenylcarbamate (**14**) (Table 2 and Fig. 7), a metabolite of a herbicide^46^, was identified from wheat bread consumption for the first time in the present study.

##### Phenolic acid derivatives

**Figure 8:**
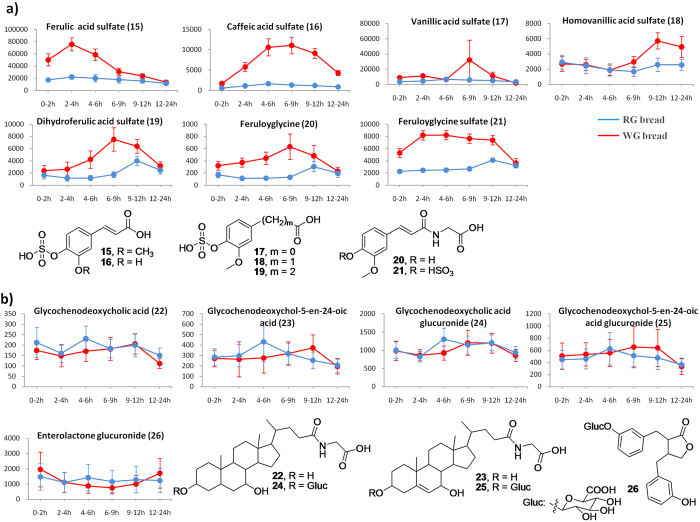
Kinetics study. Kinetics curves and structures of the main urinary metabolites derived from phenolic acids (**a**), and phytosterols and ligans (**b**), after RG- and WG bread consumptions at different intervals (0–2 h, 2–4 h, 4–6 h, 6–9 h, 9–12 h, and 12–24 h). The y-axis indicates peak area/osmolality (mOsm/kg). Data are expressed as mean ± SEM.

All phenolic acids (**15–21**) in Table 2 were identified by comparing their MS/MS fragmentation data with those given in the literature^4^,^28^,^47^. The majority of phenolics metabolites detected in urine were the end products of bacterial fermentation in the colon, such as ferulic acid (FA) sulfate (**15**), caffeic acid sulfate (**16**), and vanillic acid sulfate (**17**) (Table 2 and Fig. 8)^47^. Homovanillic acid sulfate (**18**) could also be a result of bacterial bioconversions^4^. Urinary metabolites including dihydroferulic acid (DHFA) sulfate (**19**), feruloyglycine (**20**) and feruloyglycine sulfate (**21**) (Table 2 and Fig. 8), were associated with the metabolic conversion of dietary FA by microbial enzymes or endogenous enzymes in liver^47^. Phenolic acids, such as FA, caffeic acid, and vanillic acid, are predominantly found in the outer bran layer of wheat grains^48^. This could be an explanation of the higher levels of phenolics and related metabolites in the WG group than RG group.

##### Phytosterol-related compounds

Glycine conjugated bile acids, **22–25** (Table 2 and Fig. 8), were identified by comparison of their MS/MS fragmentations with data reported previously^49^,^50^. Metabolite **25** had a molecular ion at *m/z* 622 (446+176) [M−H]^−^, which was 176 units (corresponding to a glucuronide moiety) higher than that of glycochenodeoxychol-5-en-24-oic acid (**23**), indicating **25** is a glucuronide metabolite of **23**. The MS^3^ fragmentation patterns of the major daughter ion at m/z 446 of **25** is the same as the MS^2^ spectra of metabolite **23** (Table 2), further supporting that metabolite **25** is glycochenodeoxychol-5-en-24-oic acid glucuronide, a new metabolite found in human urine. It was reported that plant sterols could be converted into bile acids such as cholic and chenodeoxycholic acids in the liver in animals and in men^51^,^52^. Bile acids are then secreted into the small intestine where they undergo several enzymatic bacterial transformations^53^, producing a large and conjugated hydrophilic bile acid pool in the host^54^. Thus, the host and gut microbiome appear to modify bile acids derived from sterols in wheat grains, that can potentially create novel conjugated bile acid profiles.

##### Lignan metabolites

Metabolite **26** had a molecular ion at *m/z* 473 (297 + 176) [M−H]^−^, suggesting that it is a conjugation of glucuronide. Daughter ions at *m/z* 253, 189, 165 and 121 in MS^3^ spectra (Table 2) agreed that the aglycone is enterolactone^55^. Therefore, metabolite **26** was recognized as enterolactone glucuronide, which is known as the mammalian fermentation product of plant lignans^56^.

#### Kinetics of urinary metabolites of WG wheat phytochemicals

The urinary changes of specific dietary exposures in two groups were indicated by kinetic curves (Figs 7 and 8) as well as fold changes at each interval (Table 2). In this study, short-, medium-, and long-term refer to half-life (t_1/2_) < 6 h, 6 h < t_1/2_ < 12 h, and 12 h < t_1/2_, respectively. Similar to 3,5-DHBA (**1**), apparent increases in other AR metabolites **2–7** in the WG group at each interval were observed (Table 2 and Fig. 7a), indicating 3,5-DHBA along with other AR metabolites, **2–7**, can be used as specific exposure biomarkers for WG wheat and rye intake. As indicated in Fig. 7a and Table 2, the urinary excretions of AR metabolites **1–7** were rapidly elevated after 2 h of administration and up to maximum around the period of 6–12 h, and thereafter decreased gradually. The levels of these metabolites were remarkably elevated in the WG group across all intervals when compared to the RG group. In particular, increases in metabolites **1–6** in the WG group were significantly different (*p* < 0.05) after the 0–2 h interval (Table 2). Moreover, the levels of metabolites **1–6** at the 12–24 h interval were still higher than those at 0–2 h (Fig. 7a), indicating the excretion of these metabolites may last longer than 24 h. All these observations featured AR metabolites **1–7** as medium- to long-term urinary biomarkers for WG wheat consumption.

In addition to HPAA sulfate (**10**), four more BX derivatives, HPAA (**9**), 2-aminophenol sulfate (**11**), HHPAA sulfate (**12**), and HHPPA sulfate (**13**) were identified in non-targeted metabolome analysis and exhibited comparable increases in the WG group at each interval, with particularly significant increases at interval of 6–9 h (*p* < 0.05) for all (Table 2 and Fig. 7b), suggesting that BX derivatives **9–13** can be used as alternative biomarkers to distinguish WG consumption over RG group. The kinetic curves indicate that BX derivatives **9–13** are excreted into urine and climbed up to maximum around 6–12 h after interventions (Fig. 7b), indicating that BX derivatives can be used as medium- to long-term urinary biomarkers to reflect WG wheat intake.

As for phenolic acids and their related compounds, elevated levels of FA sulfate (**15**), caffeic acid sulfate (**16**), DHFA sulfate (**19**), and feruloyglycine (**20**) and its sulfate (**21**) were observed at each interval in the WG group (Table 2 and Fig. 8a), suggesting that these phenolic acid metabolites may aid in discriminating WG wheat consumption from other food intake. Trajectory curves of phenolic acid derivatives showed that excretion of FA sulfate (**15**) and feruloyglycine sulfate (**21**) reached maximum concentrations in 4 h while caffeic acid sulfate (**16**), DHFA sulfate (**19**), and feruloyglycine (**20**) climbed up to maximum in 9 h (Supplementary Fig. 1 and Fig. 8a), indicating these metabolites may be short- to medium-term urinary biomarkers. Since phenoilic acids are abundant in the bran part of WG wheat^45^, these derivatives of phenolic acids may be used as biomarkers to distinguish WG consumers over RG group. However, due to non-specificities of phenolic acids in human food^57^, their derivatives can be an aid of AR metabolites and BX derivatives in measuring WG consumption in epidemiological studies.

Urinary excretion of phytosterol-related derivatives (**22**, **24**, and **25**) between WG and RG groups was not significantly different at each interval in this study, and moreover, trajectory curves of these derivatives (**22–25**) between two groups showed multiple points of intersection (Table 2 and Fig. 8b), despite the reports that glycine conjugated and unconjugated bile acids may be responsible for the discrimination between venous and arterial plasma samples after WG wheat intake in pigs^49^. Similarly, the lignan metabolite, enterolactone glucuronide (**26**), has been reported as a biomarker to distinguish WG rye consumption from RG group^39^. However, our data showed that the urinary excretion of enterolactone glucuronide in the WG group is not significantly different from the RG group (Table 2 and Fig. 8b).

As a consequence, we propose a panel of urinary biomarkers consisting of seven AR metabolites (**1–7**) and five BX derivatives (**9–13**) as more specific medium- to long-term biomarkers, as well as five phenolic acid derivatives (**15, 16, 19–21**), to estimate WG consumption (Supplementary Fig. 1; and Figs 7a,b and 8a). This combination may facilitate the objective monitoring of WG consumption for a longer period in the intervention studies.

## Discussion

In this study, a non-targeted metabolomics approach coupled with three OPLS analyses was used to investigate the metabolic difference between WG and RG samples. The results from the three analyses suggest that there was some overall difference between WG and RG samples when all of the time points were considered, but a statistically significant difference was only observed at 2–4 h and 4–6 h after the WG or RG consumption.

Endogenous differences determined by non-targeted metabolomics may reveal unexplored mechanisms responsible for the observed beneficial effects of WG consumption in relation to RG consumption in epidemiological studies, and thus provide better estimates of diet-disease associations^4^. Our results suggest that endogenous cysteine and related thiol compounds accumulate in the body after WG consumption (Table 1 and Fig. 3). Extracellular cysteine constitutes a major and independent component in antioxidant defense through cysteine/cystine redox cycle^58^. This thiol redox cycle may protect the body against oxidative stress that has been implicated in the etiology or the progression of a number of human diseases, such as cancers, CVD, and diabetes^59^. It has been revealed that cysteine/cystine redox signaling plays a vital role in preventing CVD onset or progression^60^. Recent studies have demonstrated lower blood sugar levels in normal subjects or diabetic patients after supplementation with cysteine or NAC^61^. Proteins containing oxidation-sensitive cysteines in tissue may prevent the development of tumorigenesis in colorectal cancer patients^62^. Therefore, WG wheat consumption may stimulate the antioxidant defense system to protect the body against the damage of oxidative stress when compared to RG consumption. However, additional studies are needed to further validate these findings.

A pool of endogenous metabolites related to nucleotides was extensively decreased in the WG consumers compared to the RG consumers (Table 1 and Fig. 4). Levels of urinary nucleotides, including adenine^63^, adenosine^63^,^64^, 3-methylcytidine^64^, uridine^65^,^66^, thymine^67^, Pseu^65^,^68^, m7Gua^69^, m1A^65^,^66^,^68^, t6A^66^, m22G^65^,^66^, and urate^70^, are reported to be significantly elevated in patients with various cancers. Increased urate can also be a risk factor for hypertension and CVD in patients^71^. Changes on metabolites of amino acids, such as tryptophan and proline (Table 1 and Fig. 5), are also related to risk of diseases. Indolelactate, a metabolite of tryptophan^72^, reportedly plays an important role in the progression of cancers and CVD^73^. The level of *trans*-4-hydroxyproline in cancer patients was significantly higher than those in healthy subjects^74^. Dopamine in clinical use has been proposed to have a role in the treatment of gastric cancer^75^. On the other hand, WG consumption may also produce health effects through lipid and carbohydrate metabolism (Table 1 and Fig. 5). AzA exerted antitumor properties^76^. Serum xylulose was reported to correlate to diabetics patients^77^. These results indicate that consumption of WG wheat may attenuate risk factors of cancers, CVD, and diabetes, compared to those in the RG group. However, the mechanisms underlying the protective effects of WG wheat consumption are still a future research subject.

Kinetics studies of metabolites provide detailed information of kinetic parameters, and are a better approach to validate novel metabolites as potential biomarkers for dietary exposure than those using single spot or 24-h samples in epidemiological studies. However, all of the previous metabolomics studies on WG wheat/rye investigate biomarkers of dietary exposure using merely single spot or 24-h samples^39^,^78^. This is the first study to identify the appropriate biomarkers as a panel to estimate WG wheat consumption using kinetics parameters. In the present study, the most discriminative food-derived metabolites that linked to WG wheat consumption mainly originated from ARs, BXs, and phenolic acids (Tables 1 and 2). Wheat ARs have been revealed to play an important role in the prevention of cancers^79^, diabetes^80^, and CVD^80^. Similarly, cereal BXs showed cytotoxic effects^81^, and could also significantly decrease lipid accumulation in 3T3-L1 preadipocytes^82^. In addition to the direct radical scavenging activity^83^,^84^, simple phenolic acids, such as ferulic acid, caffeic acid, gallic acid, and cinnamic acid, possess antidiabetic properties^85^. Consumption of products rich in phenolic acids correlates with a reduced risk of CVD^86^. Thus, the health-promoting effects of WG wheat consumption observed in epidemiological studies may be attributed in part to the unique phytochemical contents of ARs, BXs, and phenolic acids in this whole grain.

The fingerprinting of small molecules by non-targeted metabolomics approaches heavily relies on public databases such as HMDB, Metlin, and MassBank, and metabolites originating from specific food components are unlikely to be included in those databases. Therefore, non-targeted metabolomics approaches have limitations regarding the discovery of novel metabolites that derive from specific food components in biofluids, which could be more specific and sensitive exposure biomarkers. In contrast, targeted metabolomics is a useful tool to investigate the metabolism of specific food components using non-commercially available standards, such as synthetic products in lab and naturally occurring isolates. Therefore, a joint use of non-targeted and targeted metabolomics approaches can compensate for each other, and expedite the discovery of better biomarkers to measure WG consumption and to better predict the correlation between WG intake and the risk of chronic diseases. The biomarkers identified in this study will be used to create a library for the development of automated quantification methods to quantify these metabolites in large interventional or observational clinical studies in order to further validate the usefulness of these biomarkers. In addition, it is worthwhile to further study the mechanisms by which WG wheat intake affects the levels of these endogenous metabolites.

## Methods

### Study Design

The study design was published elsewhere^22^. In brief, twelve healthy participants had a three-day washout period to avoid foods containing phenolic compounds and all cereal bran-related products before the study and throughout the study for 5d. After the initial 3-d washout period, all participants ingested a single dose (196 g, fresh) of RG wheat bread plus 21 g of butter, and 30 min were allotted for the ingestion of the RG bread and butter. Urine samples were collected for the following 24 h in six intervals: 0–2, 2–4, 4–6, 6–9, 9–12, and 12–24 h. The following day, the same participants ingested a single dose (208 g, fresh weight) of WG wheat bread plus 21 g of butter in 30 min. In a fashion similar to RG ingestion, urine samples at six intervals in the following 24 h after WG consumption were collected. Hydrochloric acid was added to urine samples (0.4% of total urine volume). Samples were then portioned into aliquots and stored at −80 °C until analysis. Participants provided informed consent, and the study procedures were approved by the institutional review board at North Carolina Agricultural and Technical State University, Greensboro, NC (IRB#:11–0026). The experiment was conducted and data was collected according to the approved procedures at North Carolina Research Campus in Kannapolis, NC. The pharmacokinetics of several AR metabolites from these samples has been determined in our previous studies^22^. Based on our previous data, three subjects were excluded for this metabolomics study due to either high levels of AR metabolites in the RG samples or in the WG samples.

### Non-targeted Metabolomics Platform

#### Sample preparation

Sample preparation was carried out at Metabolon Inc. (Durham, NC), as described previously^87^,^88^. In brief, several recovery standards were added prior to the first step in the extraction process for quality control (QC) purposes. To remove protein, dissociate small molecules bound to protein or trapped in the precipitated protein matrix, and to recover chemically diverse metabolites, proteins were precipitated with methanol under vigorous shaking for 2 min (Glen Mills Genogrinder 2000) followed by centrifugation.

#### QA/QC

Three types of controls were analyzed in concert with the experimental samples: a pooled matrix sample generated by taking a small volume of each experimental sample as a technical replicate; extracted water samples as process blanks; and a cocktail of standards spiked into every analyzed sample for instrument performance monitoring. These QC samples and standards were described in Supplementary Tables 2 and 3. Instrument variability was determined by calculating the median relative standard deviation (RSD) for the standards that were added to each sample prior to injection into the mass spectrometers. Overall process variability was determined by calculating the median RSD for all endogenous metabolites present in 100% of the urine technical replicates. Experimental samples were randomized across the platform run, as outlined in Supplementary Fig. 3.

#### UPLC-MS/MS

The ultra-performance liquid chromatography-tandem mass spectrometry (UPLC-MS/MS) platform utilized a Waters Acquity UPLC and a Thermo Scientific Q-Exactive high resolution/accurate mass spectrometer interfaced with a heated electrospray ionization (HESI-II) source and Orbitrap mass analyzer operated at 35,000 mass resolution. The sample extract was dried then reconstituted in acidic or basic LC-compatible solvents, which contained a series of standards at fixed concentrations to ensure injection and chromatographic consistency. Two aliquots were analyzed under acidic, positive ion-optimized conditions and a third one under basic, negative ion-optimized conditions, using a Waters UPLC BEH C_18_ column (2.1 × 100 mm, 1.7 μm). Two extracts reconstituted in acidic conditions were gradient eluted using water and MeOH containing 0.05% perfluoropentanoic acid (PFPA) and 0.1% formic acid (FA) for hydrophilic compounds and MeOH, MeCN, and water containing 0.05% PFPA and 0.01% FA for hydrophobic compounds, respectively, whereas the basic extracts used water and MeOH containing 6.5 mM ammonium bicarbonate at pH 8. A fourth aliquot was analyzed via negative ionization following elution from a HILIC column (Waters UPLC BEH Amide 2.1 × 150 mm, 1.7 μm) using a gradient consisting of H_2_O and MeCN with 10 mM ammonium formate at pH 10.8. The MS analysis alternated between MS and data-dependent MS/MS scans using dynamic exclusion, and the scan range varied from 70 to 1000 *m/z*.

#### Data extraction and compound identification

Raw data was extracted, peak-identified, and QC processed. Metabolites were identified by automated comparison to library entries of purified standards. Biochemical identifications were based on three criteria: retention index (RI) within a narrow RI window of the proposed identification, accurate mass match to the library +/− 10 ppm, and the MS/MS forward and reverse scores between the experimental data and authentic standards. The MS/MS scores are based on a comparison of the ions present in the experimental spectrum to the ions present in the library spectrum.

#### Metabolite quantification and data normalization

Peaks were quantified using area-under-the-curve (AUC). For studies spanning multiple days, a data normalization step was performed to correct variation resulting from instrument inter-day tuning differences. Essentially, each compound was corrected in run-day blocks by registering the medians to equal one (1.00) and normalizing each data point proportionately (Supplementary Fig. 4). For studies that did not require more than one day of analysis, no normalization is necessary, other than data visualization. In all instances, biochemical data have been normalized to osmolality (milliosmoles of biochemicals per kg of urine, mOsm/kg) to account for differences in metabolite levels due to differences in the amount of material present in each sample.

### Targeted Metabolomics Platform

#### Sample preparation

Urine samples were prepared according to our previous method with slight modifications^22^. Briefly, 50 μL urine samples from each interval were diluted 1:1 with methanol to precipitate proteins. The mixture was vortexed for 1 min, and then centrifuged at 17,000 × g for 10 min. 50 μL of the supernatant was transferred into vials for LC-MS analysis.

#### HPLC-MS/MS

LC-MS analysis was carried out with a Thermo-Finnigan Spectra System that consists of a Dionex XRS pump, a Dionex XRS open autosampler, and a LTQ Velos Pro ion trap mass detector (Thermo Electron, San Jose, CA, USA) incorporated with an electrospray ionization (ESI) interface. A Gemini C_18_ column (150 mm × 4.6 mm i.d., 5 μm, Phenomenex) was used to analyze metabolites with a flow rate of 0.3 mL/min. The binary mobile phase system consists of 5% aqueous methanol with 0.1% formic acid (FA) as phase A and methanol with 0.1% FA as phase B. The column was eluted by a gradient progress (5% B from 0 to 7 min; 5 to 30% B from 7 to 15 min; 30–50% B from 15 to 25 min; 50–80% B from 25 to 35 min; 80–100% B from 35 to 45 min; 100% B from 45 to 55 min, and then 5% B from 55 to 60 min). The injection volume was 10 μL for each sample. The LC elute was introduced into the ESI interface. The negative ion polarity mode was set for an ESI ion source with the voltage on the ESI interface maintained at approximate 3.6 kV. Nitrogen gas was used as the sheath gas at a flow rate of 3 AU and the auxiliary gas at 10 AU, respectively. Optimized parameters include ESI interface temperature, capillary voltage, ion spray voltage, sheath gas flow rate, tube lens offset voltage, and ion optics settings.

#### Data-dependent analysis

The data-dependent experiment was set up with the following scan events: 1) scan event 1 to collect the full MS spectrum of all the ions in the sample; 2) scan event 2 to collect the MS/MS spectra of the most intense ion from the MS spectrum in scan event 1; and 3) scan event 3 collect the MS/MS spectra of the second intense ion from the MS spectrum in scan event 1. In the dynamic exclusion setting, the repeat count for each ion was 5, the repeat duration was 30 s, the exclusion list size was 50, and the exclusion duration was 180 s. The collision-induced dissociation was conducted with an isolation width of 1.0 Da and normalized collision energy of 35 for MS/MS analysis. The mass range was measured from 50 to 800 *m*/*z*. Data acquisition was performed with Xcalibur version 2.0 (Thermo Electron, San Jose, CA).

#### Identification and quantification of metabolites and data normalization

Metabolites were identified by either directly comparing the ion features in the urine samples to authentic standards in our lab or interpreting MS/MS fragmentation patterns of ions in the urines according to published data in literature. Targeted peaks were quantified using AUC. Biochemical data was normalized to the same osmolality (mOsm/kg) as used in non-targeted metabolomics analysis.

### Statistical Analysis

Data are expressed as mean ± standard error of measurement (SEM) unless otherwise stated. *P* < 0.05 was considered to indicate significance. Fold changes across intervals were calculated by dividing the mean of the normalized intensity in the WG group by the mean scaled intensity of the same compound in the RG consumption. For the non-targeted metabolomics data, raw area counts for each metabolite in each sample were normalized to osmolality. Missing values were imputed with the observed minimum after normalization. Following log transformation of the normalized data, a 2 (treatments) × 6 (time points) repeated measures ANOVA was performed to identify metabolites exhibiting significant treatment × time interaction effect. Post hoc tests were performed to compare WG and RG groups at each interval. An estimate of the false discovery rate (*q*-value) was calculated to take into account the multiple comparisons. Orthogonal Partial Least Squares Discriminant Analysis (OPLS-DA), OPLS-effect projections (OPLS-EP), and OPLS-DA based batch processing analysis in SIMCA (Umetrics, Umeå, Sweden) were used to detect a panel of metabolites that best distinguishes RG samples and WG samples. The default 7-round cross-validation in the SIMCA software package was applied with 1/7 of the samples being left out from the mathematical model in each round. Permutation-based validation was used to prevent overfitting. Variable Influence on Projection (VIP) score was calculated for each metabolite based on OPLS weight and variability explained in OPLS-EP. Metabolites with VIP >1.5 were considered important metabolites in separating WG and RG groups.

Regarding targeted metabolic profiling, raw area counts for each metabolite in each sample were normalized to the same osmolality as used in non-targeted analysis. The same repeated measures ANOVA model described above was also used to analyze the targeted metabolite data.

## Additional Information

**How to cite this article**: Zhu, Y. *et al.* Urinary Biomarkers of Whole Grain Wheat Intake Identified by Non-targeted and Targeted Metabolomics Approaches. *Sci. Rep.* **6**, 36278; doi: 10.1038/srep36278 (2016).

**Publisher’s note:** Springer Nature remains neutral with regard to jurisdictional claims in published maps and institutional affiliations.

## Supplementary Material

Supplementary Information

## Figures and Tables

**Table 1:** Most discriminative changes in humans after consuming the WG and RG diets determined by non-targeted metabolomics with an OPLS-EP analysis (VIP >1.5).

			WG versus RG (2–4 h)			WG versus RG (4–6 h)		
Metabolites^a^	Super/Sub Pathway		Fold change^b^	*p* value^c^	*q* value^d^	Fold change^b^	*p* value^c^	*q* value^d^
**Endogenous metabolites**								
Cysteine	**Amino acid**	Methionine, Cysteine, SAM and Taurine Metabolism	—	—	—	2.95	<0.001	0.008
*N*-acetylcysteine^*^			2.07	0.011	0.22	—	—	—
			0.70	0.002	0.11	0.78	0.013	0.20
Indolelactate			0.59	0.071	0.59	0.48	0.035	0.27
4-Acetamidobutanoate		Urea cycle; Arginine and Proline Metabolism	—	—	—	0.64	0.004	0.10
Imidazole lactate			—	—	—	0.63	0.028	0.25
*trans*-4-Hydroxyproline			—	—	—	0.62	0.007	0.17
*N*-Acetylarginine			—	—	—	0.58	0.041	0.30
*N*^*2*^*,N*^*5*^-Diacetylornithine			—	—	—	0.55	0.037	0.28
Argininosuccinate			0.53	0.030	0.41	0.51	0.019	0.23
Creatinine		Tryptophan Metabolism	—	—	—	0.62	0.011	0.20
5-Hydroxyindoleacetate			—	—	—	0.53	0.014	0.21
Xanthurenate			—	—	—	0.45	0.028	0.25
Dopamine sulfate (2)		Polyamine Metabolism	1.74	0.009	0.22	—	—	—
N-Acetylputrescine			—	—	—	0.63	0.015	0.21
Cysteinylglycine^*^		Glutathione Metabolism	3.04	0.009	0.22	—	—	—
Phenylacetylglutamine		Phenylalanine and Tyrosine Metabolism	—	—	—	0.57	0.015	0.21
Citramalate		Glutamate Metabolism	—	—	—	0.46	0.024	0.24
Pseudouridine	**Nucleotide**	Pyrimidine Metabolism (Uracil containing)	—	—	—	0.66	0.003	0.10
4-Ureidobutyrate			—	—	—	0.64	0.041	0.30
Uridine			—	—	—	0.61	0.027	0.25
5,6-Dihydrouracil			—	—	—	0.48	0.011	0.20
Thymine		Pyrimidine Metabolism (Thymine containing)	—	—	—	0.66	0.009	0.20
3-Methylcytidine		Pyrimidine Metabolism (Cytidine containing)	—	—	—	0.56	0.032	0.26
7-Methylguanine		Purine Metabolism (Guanine containing)	—	—	—	0.53	0.008	0.20
*N*^2^,*N*^2^-Dimethylguanosine			—	—	—	0.47	0.002	0.08
*N*^1^-Methyladenosine		Purine Metabolism (Adenine containing)	0.78	0.071	0.59	0.65	0.009	0.20
*N*^6^-Carbamoylthreonyladenosine			—	—	—	0.58	0.003	0.09
Adenosine			—	—	—	0.51	0.032	0.26
Adenine			—	—	—	0.42	0.033	0.27
Urate		Purine Metabolism (Xanthine/Inosine containing)	—	—	—	0.55	0.044	0.30
Azelate	**Lipid**	Fatty Acid, Dicarboxylate	3.07	0.014	0.26	—	—	—
Pimelate			2.26	0.014	0.26	—	—	—
Dimethylmalonic acid			1.44	0.017	0.28	—	—	—
2-Aminooctanoate		Fatty Acid, Amino	—	—	—	1.95	0.010	0.20
Ribitol	**Carbohydrate**	Pentose Metabolism	—	—	—	0.69	0.022	0.24
Ribulose/xylulose			0.59	0.029	0.41	—	—	—
*N*^1^-Methyl-2-pyridone-5-carboxamide	**Cofactors and Vitamins**	Nicotinate and Nicotinamide Metabolism	0.64	0.011	0.22	0.51	<0.001	0.02
1-Methylnicotinamide			—	—	—	0.47	0.003	0.10
Oxalate		Ascorbate and Aldarate Metabolism	0.57	0.043	0.50	—	—	—
Citraconate/glutaconate	**Energy**/TCA Cycle		2.18	<0.001	0.002	—	—	**—**
**Exogenous metabolites**								
Tartarate	**Xenobiotics**	Food Component & Plant	8.73	<0.001	0.009	21.3	<0.001	0.008
Vanillic acid			4.08	0.003	0.12	—	—	—
3,5-DHBA			3.82	<0.001	0.02	4.53	<0.001	0.01
Ferulic acid 4-sulfate			3.08	0.024	0.37	2.30	0.047	0.31
Syringic acid			2.50	0.005	0.14	—	—	—
2,3-Dihydroxyisovalerate			0.66	0.035	0.44	0.62	0.012	0.20
2-Oxindole-3-acetate			0.22	<0.001	0.006	0.27	<0.001	0.02
Gentisic acid-5-glucoside		Food Component	8.04	<0.001	0.009	3.84	<0.001	0.06
4-Vinylguaiacol sulfate			4.21	0.003	0.12	—	—	—
1,2,3-Benzenetriol sulfate (1)			2.90	0.009	0.22	2.05	0.014	0.21
3-Hydroxypyridine sulfate			2.46	0.002	0.11	1.81	0.029	0.25
1,2,3-Benzenetriol sulfate (2)			2.02	0.020	0.32	—	—	—
Lanthionine			0.65	0.055	0.57	0.61	0.024	0.24
Sulfate			—	—	—	0.60	0.012	0.20
HPAA sulfate		Food Component & Drug	4.34	<0.001	0.02	5.15	<0.001	0.008
4-Acetylphenol sulfate			2.29	0.004	0.12	—	—	—
3-Methylcatechol sulfate (2)		Benzoate Metabolism	4.97	<0.001	0.06	—	—	—
3-Methylcatechol sulfate (1)			2.78	0.004	0.12	2.15	0.019	0.23
3-Methoxycatechol sulfate (2)			1.53	0.018	0.28	2.13	0.001	0.07
1-Methylxanthine		Xanthine Metabolism	—	—	—	0.41	<0.001	0.01
1-Methylurate			—	—	—	0.57	0.015	0.21

^a^DHBA, 3,5-Dihydroxybenzoic acid; HPAA, *N*-(2-Hydroxyphenyl)acetamide.

^b^Fold change was calculated by dividing the mean of normalized intensity of each urinary metabolite after WG consumption by the mean intensity of the same urinary metabolite after RG consumption.

^c^*p* < 0.05 was assigned to be significant.

^d^*q* value was calculated for correction of false-positives. −, indicates no data found when a cut-off of >1.5 for VIP value was applied. *Indicates data (*N*-acetylcysteine and Cysteinylglycine) found only when a cut-off of >1.2 for VIP value was applied. OPLS-EP, Orthogonal Partial Least Squares-Effect Projections.

**Table 2:** Main changes in phytochemical metabolites in human urine after a wheat intervention with WG versus RG obtained by targeted metabolomics.

	RT (min)	*m/z* [M−H]^−^	MS^n^ (n = 2−4) fragmentation (*m/z*)			Fold change (WG versus RG)^d^					
				Annotation^a^		0−2 h	2−4 h	4−6 h	6−9 h	9−12 h	12−24 h
**1**	13.00	153	**153**/109	**Alkylresorcinol metabolites**	3,5-DHBA^b^,^c^	2.63 ± 0.71	6.29 ± 1.7^**^	6.09 ± 1.7^**^	12.0 ± 2.7^**^	11.6 ± 2.8^**^	8.61 ± 1.5^**^
**2**	22.16	209	**209**/191 (B), 165, 151, 123		3,5-DHPPTA^b^	1.74 ± 0.22^*^	4.65 ± 0.67^**^	6.07 ± 1.2^**^	7.96 ± 2.4^**^	7.39 ± 2.4^**^	3.28 ± 0.63^**^
**3**	10.83	210	**210**/192, 166 (B)		3,5-DHBA glycine^b^	2.05 ± 0.96	3.28 ± 0.54^*^	3.38 ± 0.82^*^	11.0 ± 2.5^**^	6.07 ± 2.5^*^	6.78 ± 1.7^**^
**4**	23.83	233	**233**/153; **153**/109		3,5-DHBA sulfate	5.23 ± 1.6^**^	6.84 ± 1.4^**^	6.35 ± 1.0^**^	9.86 ± 1.7^**^	11.4 ± 2.7^**^	9.16 ± 1.7^**^
**5**	26.22	261	**261**/181 (B), 137 **181**/163, 137(B)		3,5-DHPPA sulfate	3.06 ± 0.86^*^	7.46 ± 2.1^**^	8.86 ± 2.0^**^	8.25 ± 1.7^**^	8.01 ± 1.0^**^	4.72 ± 1.0^**^
**6**	38.41	289	**289**/209 **209**/191 (B), 165, 151, 123		3,5-DHPPTA sulfate	6.03 ± 1.7^**^	10.5 ± 2.2^**^	14.5 ± 3.8^**^	18.6 ± 5.7^**^	24.3 ± 8.8^**^	17.6 ± 8.0^**^
**7**	31.96	317	**317**/237 **237**/219 (B), 193, 177, 123 **219**/201, 177 (B), 151, 121		3,5-DHPHTA sulfate	2.58 ± 0.93	5.87 ± 1.8^*^	5.18 ± 1.2^*^	10.5 ± 4.5^**^	12.0 ± 5.4^**^	4.07 ± 1.1^*^
**8**	25.83	233	**233**/153 **153**/135, 123 (B), 109		2-(3,5-dihydroxyphenyl)- ethanol sulfate	2.19 ± 0.32^**^	1.95 ± 0.30^*^	1.74 ± 0.23^*^	2.01 ± 0.39^*^	1.23 ± 0.18	0.80 ± 0.08
**9**	17.10	150	**150**/108	**Benzoxazinoid metabolites**	HPAA	2.65 ± 1.3	3.37 ± 1.1^*^	3.73 ± 1.5	6.43 ± 1.9^**^	4.44 ± 1.1^**^	2.63 ± 0.48^*^
**10**	31.92	230	**230**/150 **150**/108		HPAA sulfate^***c***^	2.01 ± 0.61	3.73 ± 1.1^*^	8.32 ± 3.5^**^	4.39 ± 0.84^**^	2.86 ± 0.64^*^	2.82 ± 0.38^*^
**11**	8.21	188	**188**/108		2-Aminophenol sulfate	1.81 ± 0.73	1.94 ± 0.65	2.41 ± 0.59	5.71 ± 1.1^**^	1.90 ± 0.61	2.51 ± 0.37^*^
**12**	28.77	246	**246**/166 **166**/148, 118 (B), 108		HHPAA sulfate	1.22 ± 0.13^*^	1.45 ± 0.10^**^	2.18 ± 0.38^**^	1.94 ± 0.26^**^	1.37 ± 0.14^*^	1.40 ± 0.33^*^
**13**	34.12	260	**260**/180 **180**/162, 136, 118 (B), 108		HHPPA sulfate	2.31 ± 0.20	2.90 ± 0.25	5.97 ± 1.7^*^	4.33±0.75^*^	2.18 ± 0.32	2.56 ± 0.66
**14**	15.70	194	**194**/152 (B), 108		Isopropyl 2- hydroxyphenylcarbamate	1.35 ± 0.67	4.88 ± 2.9	1.16 ± 0.39	1.56 ± 0.39	3.36 ± 1.1	2.13 ± 0.70
**15**	34.29	273	**273**/229, 193 (B) **193**/178, 149 (B), 134	**Phenolic acid derivatives**	Ferulic acid sulfate^***c***^	3.01 ± 0.55^**^	3.67 ± 0.46^**^	4.05 ± 0.79^**^	2.87 ± 0.66^**^	2.51 ± 0.82^*^	1.22 ± 0.29
**16**	30.91	259	**259**/179; **179**/135		Caffeic acid sulfate	4.30 ± 1.5^**^	6.63 ± 2.5^**^	7.18±1.9^**^	8.69 ± 1.6^**^	8.33 ± 1.4^**^	5.78 ± 1.1^**^
**17**	27.96	247	**247**/203, 167 (B) **167**/152 (B), 123		Vanillic acid sulfate	2.67 ± 0.62^*^	3.13 ± 0.63^*^	2.17 ± 0.45	24.2 ± 22^*^	2.44 ± 0.93	0.69 ± 0.14
**18**	31.51	261	**261**/181 **181**/166, 137 (B)		Homovanillic acid sulfate^c^	1.07 ± 0.32	1.22 ± 0.31	1.27 ± 0.22	5.89 ± 3.0^*^	5.85 ± 2.7^**^	2.42 ± 0.55^*^
**19**	32.53	275	**275**/195 **195**/177, 151 (B), 136, 123		DHFA sulfate	1.73 ± 0.40	3.49 ± 1.6^*^	6.02 ± 3.3^**^	6.20 ± 2.7^**^	1.67 ± 0.19	1.30 ± 0.31
**20**	20.40	250	**250**/206 (B), 191, 177, 163, 149, 100		Feruloyglycine	2.10 ± 0.25^*^	3.30 ± 0.23^**^	4.02 ± 0.87^**^	5.31 ± 1.7^**^	1.64 ± 0.24^*^	1.28 ± 0.31
**21**	30.98	330	**330**/250 **250**/206 (B), 191, 177, 163, 149, 100		Feruloyglycine sulfate	2.59 ± 0.36^**^	3.42 ± 0.48^**^	3.67 ± 0.69^**^	4.41 ± 1.5^**^	1.90 ± 0.25^*^	1.29 ± 0.27
**22**	39.12	448	**448**/430, 404 (B), 386	**Phytosterol-related derivatives**	Glycochenodeoxycholic acid^c^	0.92 ± 0.16	1.31 ± 0.21	1.13 ± 0.28	0.98 ± 0.12	1.25 ± 0.24	0.95 ± 0.17
**23**	38.72	446	**446**/428, 402 (B), 384		Glycochenodeoxychol-5-en-24-oic acid	1.03 ± 0.09	1.54 ± 0.25^*^	1.53 ± 0.50	1.04 ± 0.13	1.57 ± 0.14^*^	0.99 ± 0.13
**24**	42.32	624	**624**/448 **448**/430, 404 (B), 386		Glycochenodeoxycholic acid glucuronide^c^	1.02 ± 0.15	1.09 ± 0.12	0.85 ± 0.15	1.01 ± 0.07	1.09±0.15	0.94 ± 0.09
**25**	38.61	622	**622**/446 **446**/428, 402 (B), 384		Glycochenodeoxychol-5-en-24-oic acid glucuronide	1.11 ± 0.14	1.36 ± 0.22	1.27 ± 0.36	1.06 ± 0.14	1.33 ± 0.17	0.96 ± 0.14
**26**	28.99	473	**473**/297 **297**/253 (B), 189, 165, 121	**Lig**	Enterolactone glucuronide	2.37 ± 0.80	1.78 ± 0.44	1.60±0.52	1.96 ± 0.57	1.60 ± 0.41	1.79 ± 0.49

^a^DHBA, 3,5-Dihydroxybenzoic acid; DHPPA, 3-(3,5-Dihydroxyphenyl)propanoic acid; DHPPTA, 5-(3,5-Dihydroxyphenyl)pentanoic acid; DHPHTA, 7-(3,5-Dihydroxyphenyl)heptanoic acid; DHFA, Dihydroferulic acid; HPAA, *N*-(2-Hydroxyphenyl)acetamide; HHPAA, 2-Hydroxy-*N*-(2-hydroxyphenyl)acetamide; HHPPA, 3-Hydroxy-*N*-(2-hydroxyphenyl)propanamide; Lig, Lignans.

^b^Identified using authentic standards.

^c^Identified also by non-targeted metabolomics.

^d^Fold change was calculated by dividing the mean of normalized intensity of each urinary metabolite after WG intake by the mean of normalized intensity of the same urinary metabolite after RG treatment. Data are expressed as mean ± SEM. A repeated measures ANOVA analysis was carried out to decide metabolites that differed significantly between experimental groups. ^*^p < 0.05; ^**^p < 0.001.
