# Supplementary material for: Urinary Biomarkers of Whole Grain Wheat Intake Identified by Non-targeted and Targeted Metabolomics Approaches
Source: Sci Rep. 2016 Nov 2;6:36278. doi: 10.1038/srep36278 (PMC5090248; doi:10.1038/srep36278)
Supplement: Supplementary Information [file srep36278-s1.pdf]

Supplementary Information

Urinary Biomarkers of Whole Grain Wheat Intake Identified by Non-targeted and Targeted Metabolomics Approaches

Yingdong Zhu[1], Pei Wang[1], Wei Sha[2], Shengmin Sang[1,*]

[1]Laboratory for Functional Foods and Human Health, Center for Excellence in Post-Harvest Technologies, North Carolina Agricultural and Technical State University, North Carolina Research Campus, Kannapolis, NC, USA

[2]Bioinformatics Services Division, University of North Carolina at Charlotte, North Carolina Research Campus, Kannapolis, NC, USA

Running Title: Biomarkers of Whole Grain Wheat Intake

[1 *] Correspondence and requests for materials should be addressed to S.S. (ssang@ncat.edu or shengminsang@yahoo.com).

SUPPLEMENTARY FIGURE LEGENDS

Supplementary Figure 1. Kinetics study. Kinetic curves of the 43 metabolites listed in Table 1 that are not included in Figs 3-5. Data are expressed as mean ± SEM.

Supplementary Figure 2. OPLS-DA plots from the batch processing analysis for the separation between RG samples and WG samples when all time points were considered. (**a**) OPLS-DA score scatter plots of RG samples (blue squares) and WG samples (red squares), where samples from different time points (0-2, 2-4, 4-6, 6-9, 9-12, and 12-24h) for each subject within each treatment were depicted as a single coordinate. (**b**) The OPLS-DA validation plot for the separation observed in (**a**). Q^2Y from the original data (green) was not found to be higher than 95% of the permutated data (blue), therefore this model did not pass permutation based validation.

Supplementary Figure 3. Preparation of client-specific technical replicates. A small aliquot of each client sample (colored cylinders) is pooled to create a CMTRX technical replicate sample (multi-colored cylinder), which is then injected periodically throughout the platform run. Variability among consistently detected biochemicals can be used to calculate an estimate of overall process and platform variability.

Supplementary Figure 4. Visualization of data normalization steps for a multiday platform run.

SUPPLEMENTARY TABLE LEGENDS

Supplementary Table 1. Most discriminative changes in humans after consuming the WG and RG diets determined by non-targeted metabolomics with an OPLS-EP analysis (VIP > 1.2).

Supplementary Table 2. Description of Metabolon QC Samples.

Supplementary Table 3. Metabolon QC Standards.

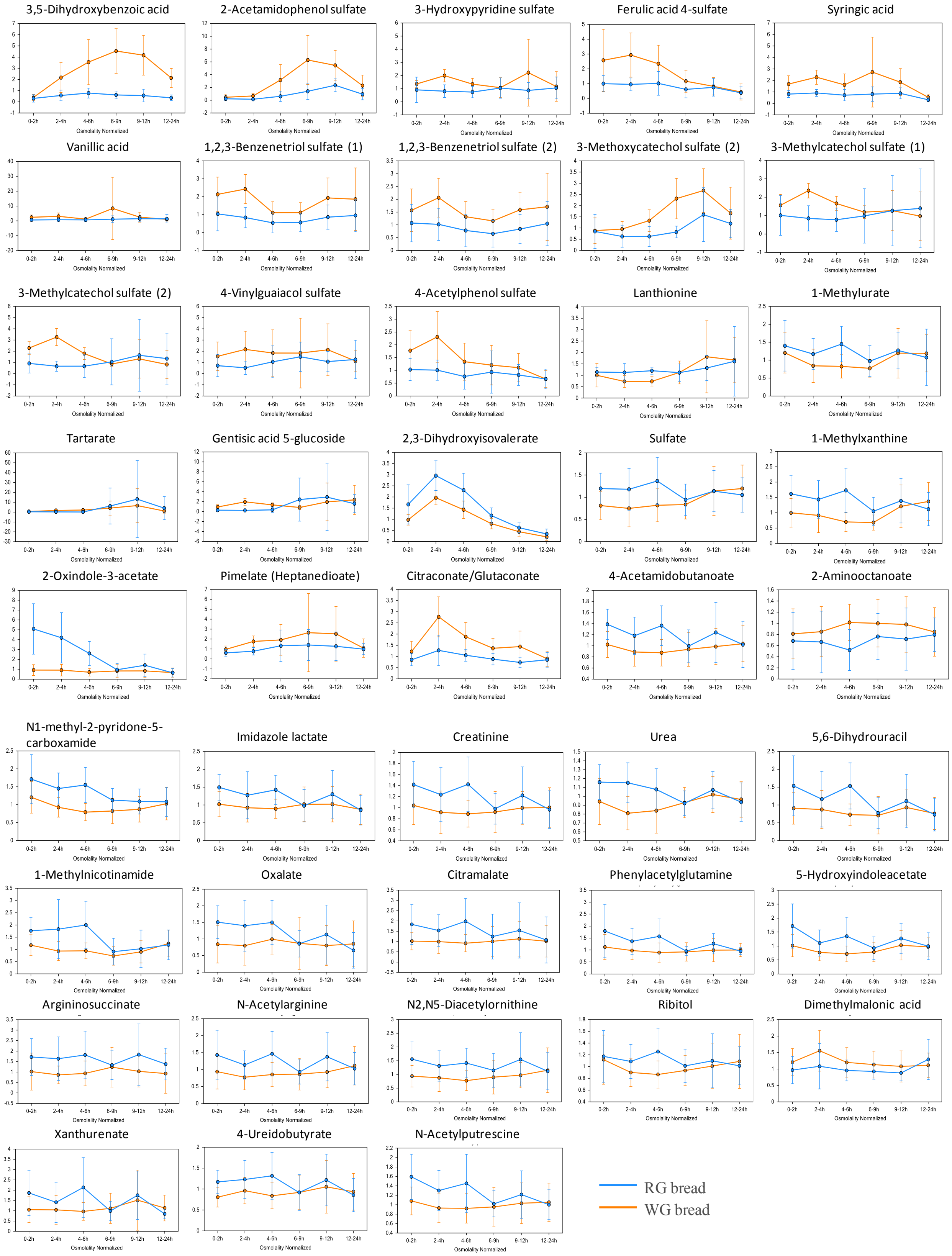
3,5-Dihydroxybenzoic acid
2-Acetamidophenol sulfate
3-Hydroxypyridine sulfate
Ferulic acid 4-sulfate
Syringic acid
Vanillic acid
1,2,3-Benzenetriol sulfate (1)
1,2,3-Benzenetriol sulfate (2)
3-Methoxycatechol sulfate (2)
3-Methylcatechol sulfate (1)
3-Methylcatechol sulfate (2)
4-Vinylguaiacol sulfate
4-Acetylphenol sulfate
Lanthionine
1-Methylurate
Tartarate
Gentisic acid 5-glucoside
2,3-Dihydroxyisovalerate
Sulfate
1-Methylxanthine
2-Oxindole-3-acetate
Pimelate (Heptanedioate)
Citraconate/Glutaconate
4-Acetamidobutanoate
2-Aminooctanoate
N1-methyl-2-pyridone-5-carboxamide
Imidazole lactate
Creatinine
Urea
5,6-Dihydrouracil
1-Methylnicotinamide
Oxalate
Citramalate
Phenylacetylglutamine
5-Hydroxyindoleacetate
Argininosuccinate
N-Acetylarginine
N2,N5-Diacetylornithine
Ribitol
Dimethylmalonic acid
Xanthurenate
4-Ureidobutyrate
N-Acetylputrescine
Osmolality Normalized
0-2h
2-4h
4-6h
6-9h
9-12h
12-24h
RG bread
WG bread

S4

Supplementary Figure 1. Kinetics study. Kinetic curves of the 43 metabolites listed in Table 1 that are not included in Figs 3-5. Data are expressed as mean ± SEM. The y-axis indicates area counts/osmolality (mOsm/kg).

a)

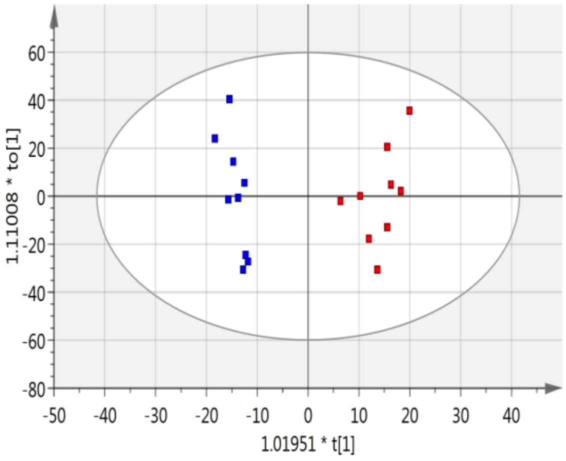

b)

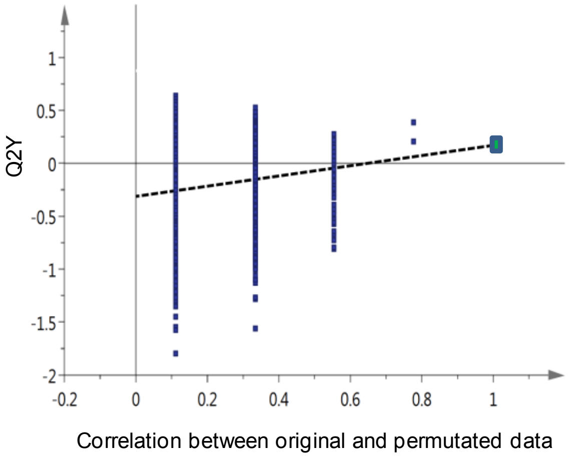

Supplementary Figure 2. OPLS-DA plots from the batch processing analysis for the separation between RG samples and WG samples when all time points were considered. (**a**) OPLS-DA score scatter plots of RG samples (blue squares) and WG samples (red squares), where samples from different time points (0-2, 2-4, 4-6, 6-9, 9-12, and 12-24h) for each subject within each treatment were depicted as a single coordinate. (**b**) The OPLS-DA validation plot for the separation observed in (**a**). Q^2Y from the original data (green) was not found to be higher than 95% of the permutated data (blue), therefore this model did not pass permutation based validation.

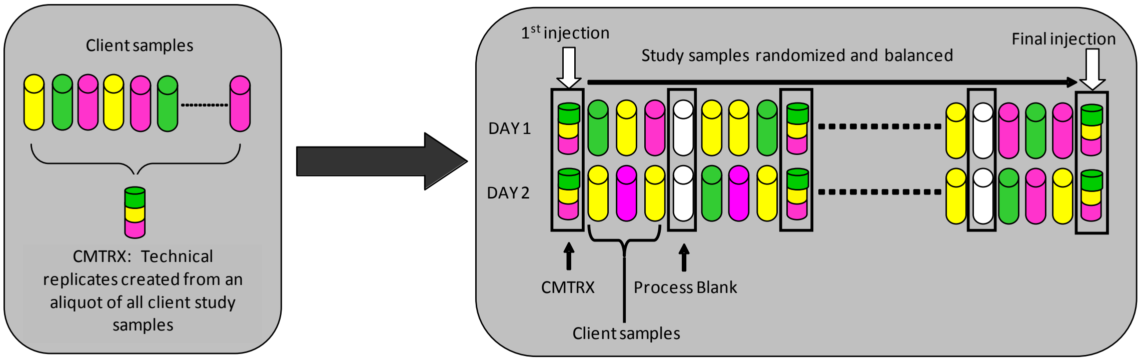

Supplementary Figure 3. Preparation of client-specific technical replicates. A small aliquot of each client sample (colored cylinders) is pooled to create a CMTRX technical replicate sample (multi-colored cylinder), which is then injected periodically throughout the platform run. Variability among consistently detected biochemicals can be used to calculate an estimate of overall process and platform variability.

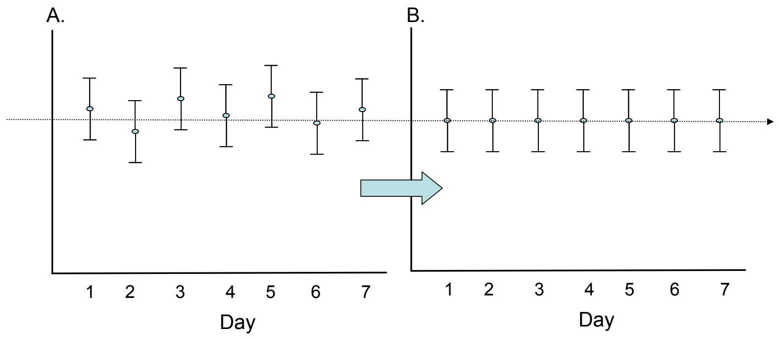

Supplementary Figure 4. Visualization of data normalization steps for a multiday platform run.

Supplementary Table 1. Most discriminative changes in humans after consuming the WG and RG diets determined by non-targeted metabolomics with an OPLS-EP analysis (VIP > 1.2).

BIOCHEMICAL	Super Pathway	Sub_Pathway	WG versus RG (2-4h)				WG versus RG (4-6h)			
			VIP [a]	Fold change [b]	p value [c]	q value [d]	VIP [a]	Fold change [b]	p value [c]	q value [d]
**Endogenous metabolites**										
vanillic alcohol sulfate	Amino Acid	Phenylalanine and Tyrosine Metabolism	1.47	2.05	0.014	0.26	0.18	0.73	0.12	0.51
dopamine sulfate (2)			1.81	1.74	0.009	0.22	0.52	1.13	0.65	0.91
dopamine sulfate (1)			1.47	1.81	0.026	0.38	1.29	1.30	0.25	0.67
4-hydroxyphenylpyruvate			1.45	1.76	0.033	0.43	1.04	1.33	0.14	0.54
Phenylacetylglutamate			1.50	0.59	0.082	0.61	1.39	0.51	0.039	0.29
Phenylacetylglutamine			1.62	0.72	0.14	0.67	1.68	0.57	0.015	0.21
Indolelactate		Tryptophan Metabolism	1.87	0.59	0.071	0.59	1.64	0.48	0.035	0.27
5-hydroxyindoleacetate			1.38	0.70	0.11	0.65	1.57	0.53	0.014	0.21
Kynurenate			1.08	0.77	0.19	0.78	1.47	0.56	0.017	0.22
Xanthurenate			0.68	0.74	0.38	0.91	1.51	0.45	0.028	0.25
Argininosuccinate		Urea cycle; Arginine and Proline Metabolism	1.54	0.53	0.030	0.41	1.64	0.51	0.019	0.23
N2,N5-diacetylornithine			1.74	0.67	0.098	0.64	1.72	0.55	0.037	0.28
N-acetylarginine			1.54	0.68	0.069	0.59	1.63	0.58	0.041	0.30
Urea			1.58	0.70	0.002	0.11	1.52	0.78	0.013	0.20
trans-4-hydroxyproline			1.56	0.77	0.13	0.66	1.64	0.62	0.007	0.17
Cysteinylglycine		Glutamate Metabolism	1.28	3.04	0.010	0.22	1.18	2.66	0.012	0.20
Citramalate			1.41	0.65	0.11	0.65	1.62	0.46	0.024	0.24
gamma-carboxyglutamate			1.38	0.80	0.06	0.59	1.02	0.77	0.028	0.25
Cysteine		Methionine, Cysteine, SAM and Taurine Metabolism	1.37	2.33	0.001	0.071	1.50	2.95	<0.001	0.008
Cystine			1.47	0.55	0.031	0.41	1.16	0.73	0.16	0.58
N-acetylcysteine			1.38	2.07	0.011	0.22	1.02	1.64	0.017	0.22
N-acetylputrescine		Polyamine Metabolism	1.47	0.71	0.052	0.55	1.56	0.63	0.015	0.21
4-acetamidobutanoate			1.40	0.75	0.055	0.57	1.67	0.64	0.004	0.10
imidazole lactate		Histidine Metabolism	1.22	0.72	0.14	0.69	1.69	0.63	0.028	0.25
N-acetyl-1-methylhistidine*			0.63	0.77	0.39	0.92	1.38	0.41	0.048	0.32
N-acetyltaurine		Methionine, Cysteine, SAM and Taurine Metabolism	1.15	0.81	0.12	0.66	1.28	0.69	0.003	0.092
methionine sulfone			0.76	0.94	0.59	0.98	1.48	0.78	0.047	0.31
sarcosine (N-Methylglycine)		Glycine, Serine and Threonine Metabolism	1.13	1.32	0.28	0.86	1.39	2.24	0.031	0.25

Creatinine		Creatine Metabolism	1.32	0.74	0.064	0.59	1.68	0.62	0.011	0.20
5,6-dihydrouracil	Nucleotide	Pyrimidine Metabolism, Uracil containing	0.81	0.75	0.24	0.83	1.75	0.48	0.011	0.20
5-methyluridine (ribothymidine)			0.83	1.36	0.29	0.88	1.37	1.95	0.030	0.25
3-ureidopropionate			0.91	0.90	0.53	0.96	1.43	0.70	0.025	0.24
Uridine			1.29	0.59	0.030	0.41	1.54	0.61	0.027	0.25
Pseudouridine			1.13	0.79	0.12	0.66	1.54	0.66	0.003	0.10
Uracil			1.40	0.70	0.10	0.64	1.47	0.60	0.015	0.21
N3-methyluridine			1.53	0.73	0.073	0.60	1.43	0.63	0.012	0.20
4-ureidobutyrate			1.11	0.78	0.25	0.84	1.57	0.64	0.041	0.30
Cytidine		Pyrimidine Metabolism, Cytidine containing	1.31	0.53	0.039	0.47	1.55	0.52	0.10	0.47
3-methylcytidine			1.23	0.65	0.09	0.63	1.68	0.56	0.032	0.26
Thymine		Pyrimidine Metabolism, Thymine containing	1.05	0.83	0.29	0.88	1.52	0.66	0.009	0.20
Urate		Purine Metabolism, (Hypo)Xanthine/Inosine containing	1.43	0.59	0.029	0.41	1.52	0.55	0.044	0.30
Xanthine			0.86	0.69	0.11	0.65	1.46	0.48	0.025	0.24
Adenine		Purine Metabolism, Adenine containing	1.06	0.52	0.083	0.61	1.64	0.42	0.033	0.27
Adenosine			0.98	0.58	0.068	0.59	1.67	0.51	0.032	0.26
N6-carbamoylthreonyladenosine			1.42	0.73	0.058	0.59	1.63	0.58	0.003	0.092
N1-methyladenosine			1.45	0.78	0.071	0.59	1.56	0.65	0.009	0.20
N2,N2-dimethylguanosine		Purine Metabolism, Guanine containing	1.26	0.64	0.036	0.44	1.71	0.47	0.002	0.083
7-methylguanine			1.09	0.67	0.051	0.55	1.71	0.53	0.008	0.20
azelate (nonanedioate)	Lipid	Fatty Acid, Dicarboxylate	1.76	3.077	0.014	0.26	1.22	2.36	0.059	0.36
pimelate (heptanedioate)			1.76	2.26	0.014	0.26	0.74	1.44	0.10	0.48
dimethylmalonic acid			1.79	1.44	0.017	0.28	1.18	1.26	0.21	0.62
2-aminooctanoate		Fatty Acid, Amino	1.10	1.28	0.21	0.80	1.59	1.95	0.010	0.20
Mevalonate		Mevalonate Metabolism	1.25	2.11	0.008	0.22	0.48	1.09	0.58	0.89
trimethylamine N-oxide		Phospholipid Metabolism	1.33	0.54	0.017	0.28	1.15	0.65	0.035	0.27
Carnitine		Carnitine Metabolism	1.37	0.61	0.045	0.52	1.33	0.56	0.024	0.24
cortisol glucuronide		Steroid	0.012	0.99	0.66	0.98	1.47	1.85	0.021	0.24
Arabinose	Carbohydrate	Pentose Metabolism	1.46	4.49	0.004	0.13	1.19	3.21	0.0267	0.25
Xylose			1.47	3.73	0.017	0.28	1.44	7.58	0.011	0.20
ribulose/xylulose			1.55	0.59	0.029	0.41	1.42	0.52	0.013	0.20
Ribitol			1.77	0.83	0.23	0.83	1.59	0.69	0.022	0.24
erythronate*		Aminosugar Metabolism	1.08	0.87	0.21	0.79	1.41	0.74	0.045	0.31

N-acetylneuraminate			0.81	0.90	0.44	0.92	1.35	0.76	0.046	0.31
N-acetylglucosaminylasparagine			1.06	0.86	0.26	0.86	1.27	0.71	0.017	0.22
1-methylnicotinamide	Cofactors and Vitamins	Nicotinate and Nicotinamide Metabolism	1.26	0.51	0.010	0.22	1.56	0.47	0.003	0.10
N1-Methyl-2-pyridone-5-carboxamide			1.48	0.64	0.011	0.22	1.63	0.51	<0.001	0.025
Nicotinamide			0.94	0.53	0.085	0.61	1.42	0.48	0.043	0.30
nicotinamide N-oxide			1.46	0.54	0.082	0.61	1.42	0.45	0.044	0.30
riboflavin (Vitamin B2)		Riboflavin Metabolism	1.45	0.26	0.002	0.11	1.27	0.22	0.002	0.083
oxalate (ethanedioate)		Ascorbate and Aldarate Metabolism	1.58	0.57	0.043	0.50	1.44	0.66	0.32	0.73
Pterin		Pterin Metabolism	1.14	0.49	0.087	0.62	1.43	0.32	0.029	0.25
gamma-glutamyltyrosine	Peptide	Gamma-glutamyl Amino Acid	0.45	1.26	0.35	0.89	1.37	2.39	0.035	0.27
Phenylalanylglycine		Dipeptide	1.43	0.73	0.080	0.61	1.47	0.67	0.020	0.23
citraconate/glutaconate	Energy	TCA Cycle	1.74	2.18	<0.001	0.002	1.39	1.785	0.001	0.069
**Food-derived metabolites**										
N-acetylpyrraline	Xenobiotics	Food Component/Plant	1.29	8.69	0.002	0.11	1.14	4.89	0.007	0.17
2-oxindole-3-acetate			1.68	0.22	<0.001	0.006	1.75	0.27	<0.001	0.016
Vanillate			1.70	4.087	0.003	0.12	1.32	1.78	0.075	0.39
ferulic acid 4-sulfate			1.75	3.087	0.024	0.37	1.48	2.30	0.047	0.31
4-vinylguaiacol sulfate			1.66	4.21	0.003	0.12	0.92	1.75	0.069	0.38
Tartarate			1.85	8.73	<0.001	0.009	1.69	21.3	<0.001	0.008
syringic acid			1.86	2.50	0.005	0.14	1.45	2.24	0.001	0.071
3,5-dihydroxybenzoic acid			1.55	3.82	<0.001	0.017	1.57	4.53	<0.001	0.013
Pyrraline			1.29	61.0	0.003	0.11	0.80	9.83	0.49	0.84
2,3-dihydroxyisovalerate			1.71	0.66	0.035	0.44	1.63	0.62	0.012	0.20
Homocitrate			0.47	0.77	0.81	0.98	1.34	1.99	0.018	0.23
3-hydroxypyridine sulfate		Food Component	1.77	2.46	0.002	0.11	1.52	1.81	0.029	0.25
1,2,3-benzenetriol sulfate (2)			1.67	2.02	0.020	0.32	1.40	1.69	0.043	0.30
Lanthionine			1.55	0.65	0.055	0.57	1.74	0.61	0.024	0.24
gentisic acid-5-glucoside			1.84	8.04	<0.001	0.009	1.68	3.84	<0.001	0.055
1,2,3-benzenetriol sulfate (1)			1.80	2.90	0.010	0.22	1.56	2.05	0.014	0.21
sulfate*			1.27	0.63	0.010	0.22	1.50	0.60	0.012	0.20
O-sulfo-L-tyrosine			1.38	0.82	0.12	0.65	0.99	0.75	0.025	0.24
2-aminophenol sulfate			1.06	1.26	0.39	0.92	1.36	2.52	0.004	0.10
3-methyl catechol sulfate (1)		Benzoate Metabolism	1.78	2.78	0.004	0.12	1.51	2.15	0.019	0.23
3-methyl catechol sulfate (2)			1.88	4.97	<0.001	0.06	1.35	2.71	0.002	0.092
3-methoxycatechol sulfate (2)			1.55	1.53	0.018	0.28	1.51	2.13	0.001	0.069
4-hydroxyhippurate			1.128	0.83	0.18	0.73	1.40	0.69	0.020	0.23
4-acetylphenol sulfate		Food Component & Drug	1.78	2.29	0.004	0.12	1.42	1.76	0.011	0.20
2-acetamidophenol sulfate			1.62	4.34	<0.001	0.02	1.49	5.15	<0.001	0.008

1-methylxanthine		Xanthine Metabolism	1.32	0.64	0.015	0.28	1.63	0.41	<0.001	0.013
1-methylurate			1.19	0.72	0.096	0.64	1.55	0.57	0.015	0.21

[a] VIP, Variability Influence on Projection. [b] Fold change was calculated by dividing the mean of normalized intensity of each urinary metabolite after WG consumption by the mean intensity of the same urinary metabolite after RG consumption. [c] $p < 0.05$ was assigned to be significant. [d] q value was calculated for correction of false-positives. OPLS-EP, Orthogonal Partial Least Squares-Effect Projections.

Supplementary Table 2. Description of Metabolon QC Samples

Type	Description	Purpose
MTRX	Large pool of human plasma maintained by Metabolon that has been characterized extensively.	Assure that all aspects of the Metabolon process are operating within specifications.
CMTRX	Pool created by taking a small aliquot from every customer sample.	Assess the effect of a non-plasma matrix on the Metabolon process and distinguish biological variability from process variability.
PRCS	Aliquot of ultra-pure water	Process Blank used to assess the contribution to compound signals from the process.
SOLV	Aliquot of solvents used in extraction.	Solvent Blank used to segregate contamination sources in the extraction.

Supplementary Table 3. Metabolon QC Standards

Type	Description	Purpose
RS	Recovery Standard	Assess variability and verify performance of extraction and instrumentation.
IS	Internal Standard	Assess variability and performance of instrument.
